# Information set supported deep learning architectures for improving noisy image classification

**DOI:** 10.1038/s41598-023-31462-6

**Published:** 2023-03-17

**Authors:** Saurabh Bhardwaj, Yizhi Wang, Guoqiang Yu, Yue Wang

**Affiliations:** 1 Department of Electrical and Instrumentation Engineering, Thapar Institute of Engineering and Technology, Patiala, Punjab 147004 India; 2 Department of Electrical and Computer Engineering, Virginia Polytechnic Institute and State University, Arlington, VA 22203 USA

**Keywords:** Computational science, Computer science

## Abstract

Deep learning models have been widely used in many supervised learning applications. However, these models suffer from overfitting due to various types of uncertainty with deteriorating performance when facing data biases, class imbalance, or noise propagation. The Information-Set Deep learning (ISDL) architectures with four variants are developed by integrating information set theory and deep learning principles to address the critical problem of the absence of robust deep learning models. There is a description of the ISDL architectures, learning algorithms, and analytic workflows. The performance of the ISDL models and standard architectures is evaluated using a noise-corrupted benchmark dataset. The experimental results show that the ISDL models can efficiently handle noise-dominated uncertainty and outperform peer architectures.

## Introduction

Although Deep Learning Neural Networks (DL-NN) are efficient at classifying images, noise in either training or testing dataset propagates through the layers of DL-NN or Convolutional Neural Network (CNN) and significantly deteriorates the performance of these models. The information-set deep learning (ISDL) architectures are introduced here for handling noisy data applicable to broad supervised learning tasks. To develop noise-resistant deep learning models, researchers employed a variety of approaches, including modifications to the architecture^1^, regularization methods^2–4^, and in the loss functions^5^. In some studies, image restoration was tried to restore clear latent images from corrupted observations. The researchers trained the state-of-the-art deep neural networks on images from the original dataset without noise and then used to classify images degraded by noise^6^. It has been shown that the performance of state-of-the-art DL-NN decreases when classifying low-quality images.

The concept of information set was first introduced by Hanmandlu et al.^7^ based on an entropy framework, mainly aimed to address the limitations of fuzzy set theory^8^. The potential of entropy-based feature extraction approaches is underutilized compared to conventional techniques like PCA, ICA, LDA, etc. Information set theory has been proven to be highly effective for addressing uncertainty and achieving superior performance in various settings. The integration of the information set with deep learning models is proposed to leverage this capability in deep learning models. The ISDL architectures are general and broadly applicable to many deep-learning tasks. In classic fuzzy set theory, membership functions (MF) play central roles while original signals (information source) and their potential interactions with fuzzy memberships are primarily ignored^3^. Since the MF value only measures the extent to which an information source value belongs to the set, it cannot accurately express the overall uncertainty pertaining to all information source values. To address this limitation, the information set is formulated based on an entropy framework and used to modulate fuzzy memberships by some form of the original signal^7,9,10^. The Pal and Pal entropy^11^ has been extended to an information-theoretic entropy structure and further established into information-set theory by subsequent improvements^9,10,12–16^.

In the past, the Information-set theory has been exploited and integrated into many machine-learning models to develop various features and classifiers in noisy environment. The ability of information set theory to represent probabilistic uncertainty and possibilistic certainty is described by Grover et al.^17^. Six features are created for the face recognition application Sayeed et al.^18^ utilizing the information set, the Hanman Filter (HF), and the Hanman Transforms (HT). The HF is designed to adjust the information values using a cosine function. In contrast, the HT is designed to evaluate the information source values based on the information obtained from them. The new features based on higher-order information sets are developed by Grover et al.^19^, which use fewer features per sample and have a lower time complexity than the most recent features. It was demonstrated that these features could accurately represent the multispectral palmprints. In addition to feature extraction, an information processing-based fuzzy classifier is also developed. The evolutionary method known as Human effort for achieving the goal (HEFAG), which is based on the human approach to learning and does not require algorithmic specific parameters, was developed by using information set theory^20^. The two-fold information set (TFIS) features for text-independent speaker identification and gate recognition is developed^12,16^. The entropy framework creates the TFIS features, which capture spatial and temporal information components. The TFIS features are fewer in number, which reduces computing complexity and time. These features boost performance in noisy environments. Moreover, the so-called swish activation function, recently proposed by google brain, exhibits improved performance in deep learning models, particularly in image classification and machine translation^21^. With a closer inspection of both formulation and experimental results, it is found that the swish activation function also has some roots in information-set theory.

The Infor-Set Deep Feedforward Networks (ISNN) and Infor-Set Convolutional Neural Networks (ISCNN) and their variants are the two Infor-Set supported Deep Learning architectures that are proposed and described here. ISNN employs the Infor-Layer, which is applied both after the source and after each dense layer. The Convolutional Neural Network (CNN) is altered in ISCNN by adding the Infor-Layer and/or by swapping the Infor-pool for the Pooling layer. The key benefit of the proposed models is that they perform well on noisy samples without any additional pre-processing after being trained on clean samples. The various high-level features corresponding to the CNN layers are enhanced with the aid of the Infor-layer and/or Infor-Pool layer. In the current work, an information-set layer (Infor-Layer) and a pooling method (Infor-Pool) are proposed by using information sets that are further integrated with prominent deep learning designs to improve the performance of deep learning architectures. The Infor-Layer is added after the input and between the standard layers to extract effective information and advance to deeper representations. In contrast, the Infor-Pool acquires localized information and reduces dimensionality. These specifically designed information set layers and pooling methods improve the noise robustness of classic deep learning models.

The effectiveness and robustness of the two ISDL architectures and standard models are assessed by using two independent benchmark datasets that have been degraded by noise. To show how effective the suggested Infor-layer and Infor-Pool layers are, these reformulated layers are added to the classic CNN designs, and the performance is compared to that of the architectures without them. The experimental results show that the proposed ISDL architectures can efficiently handle uncertainty and related issues and achieve superior performance compared to peer methods, where the data are corrupted with the noise of varying Peak Signal-To-Noise Ratio (PSNR).

## Results

### Infor-set based deep learning (ISDL)

When the input data are affected by noise, the information set theoretically measures the quality of contaminated attribute values in terms of possibilistic uncertainty^3^. Based on this interpretation, the ISNN and ISCNN are proposed to boost the classification performances.

Multiple evaluation criteria, including Accuracy, Precision, Recall, F1 score, and ROC-AUC, are utilized to assess and compare the performance of proposed networks with that of conventional deep networks (Supplementary Information). Performance robustness against noise is evaluated by using both the noise-corrupted MNIST dataset of handwritten digits and the EMNIST Balanced dataset with varying Peak Signal-To-Noise Ratio (PSNR). The EMNIST Balanced dataset is an extended and comprehensive collection of both handwritten characters and handwritten digits. It extends the classic MNIST dataset by incorporating a more diverse set of pattern classes. The dataset encompasses 47 unique classes of characters and digits, comprising both upper and lower-case alphabetical letters and the digits 0 to 9.

The PSNR is an expression for the ratio between a signal's maximum possible value (power) and the power of distorting noise that influences its representation quality. Mathematically, for a noise-free $m\times n$ monochrome image ‘I’ and its noisy version ‘K’ it can be represented as

1 $$PSNR = 20 \log_{10} \left( {\frac{{MAX_{I} }}{{\sqrt {MSE} }}} \right)$$

where,

$$MSE = { }\frac{1}{mn}\mathop \sum \limits_{i = 0}^{m - 1} \mathop \sum \limits_{j = 0}^{n - 1} \left[ {I\left( {i,j} \right) - K\left( {i,j} \right)} \right]^{2}$$

$$MAX_{I} = {\text{Maximum}}\;{\text{possible}}\;{\text{pixel}}\;{\text{value}}\;{\text{of}}\;{\text{the}}\;{\text{image}}$$

### Infor-set based convolutional neural network (ISCNN)

**Figure 1:**
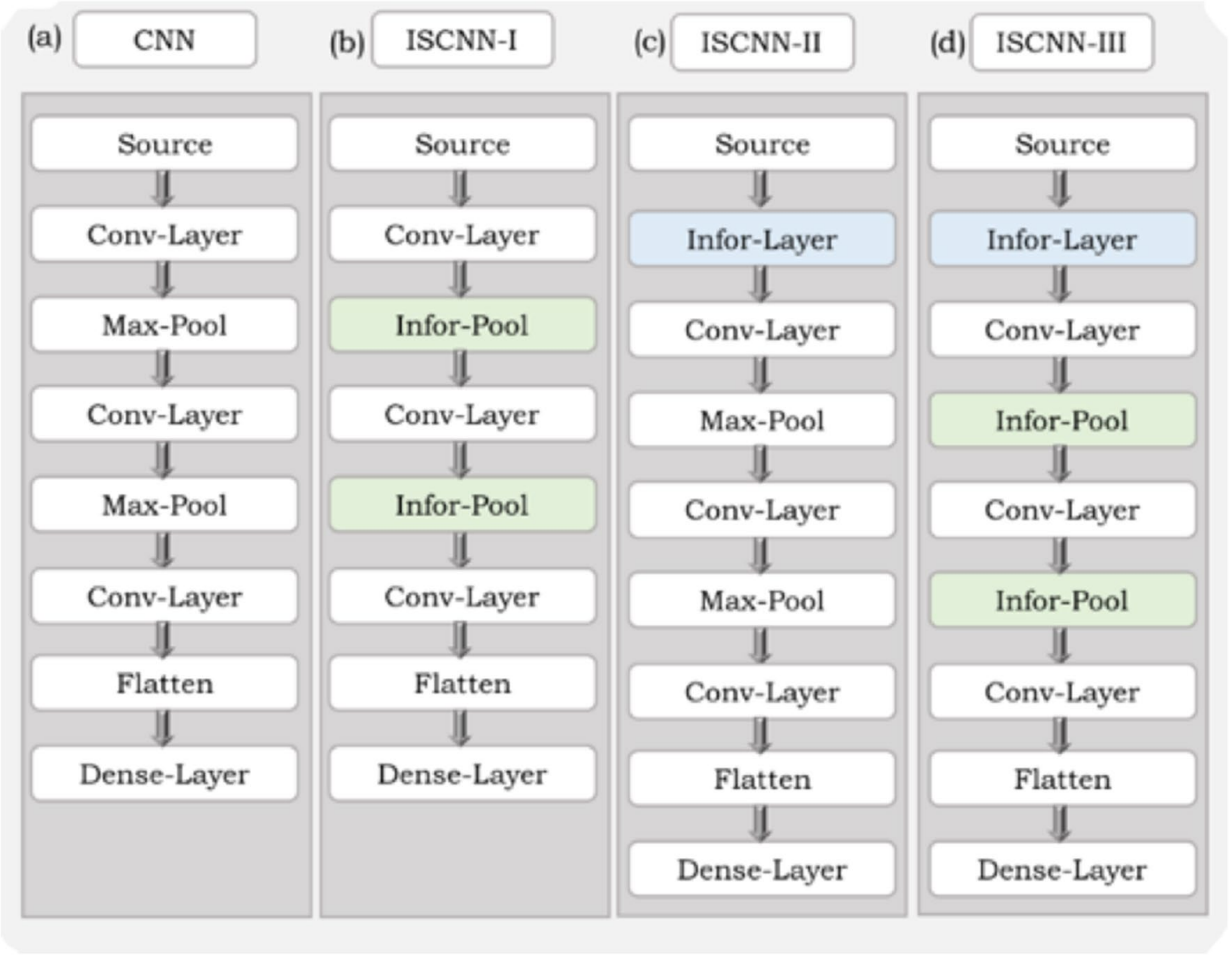
Different architectures of Information Set based Convolutional Neural Network (**a**) Structure of baseline Convolutional Neural Network (**b**) ISCNN-I: First variant of CNN in which the Max-Pool layer is replaced with Infor-Pool layer (**c**) ISCNN-II: Second variant of CNN in which the input layer is not directly connected to the Conv-Layer instead it is connected through Infor-Layer (**d**) ISCNN-III: Third variant of CNN which consists of both the Infor-Layer and Infor-Pool Layer.

Three variants of the ISCNN architectures, namely ISCNN-I, ISCNN-II, and ISCNN-III are proposed, as shown in Fig. 1. The proposed variants modify the CNN by introducing the Infor-Layer and/or replacing the Pooling layer with the Infor-pool. Figure 1 demonstrates one of the possible modifications in the CNN architecture(s). However, the Infor-layer and Infor-Pool can be introduced at different places in the network architecture.

In ISCNN-I, the Pooling layer is replaced with Infor-Pool to extract the localized information and reduce the dimensionality. Whereas in ISCNN-II, the input features are not directly connected to the convolution block; instead, the Infor-Layer is introduced before the convolution block to extract effective information of the signal of interest. ISCNN-III is the fusion of ISCNN-I and ISCNN-II.

**Figure 2:**
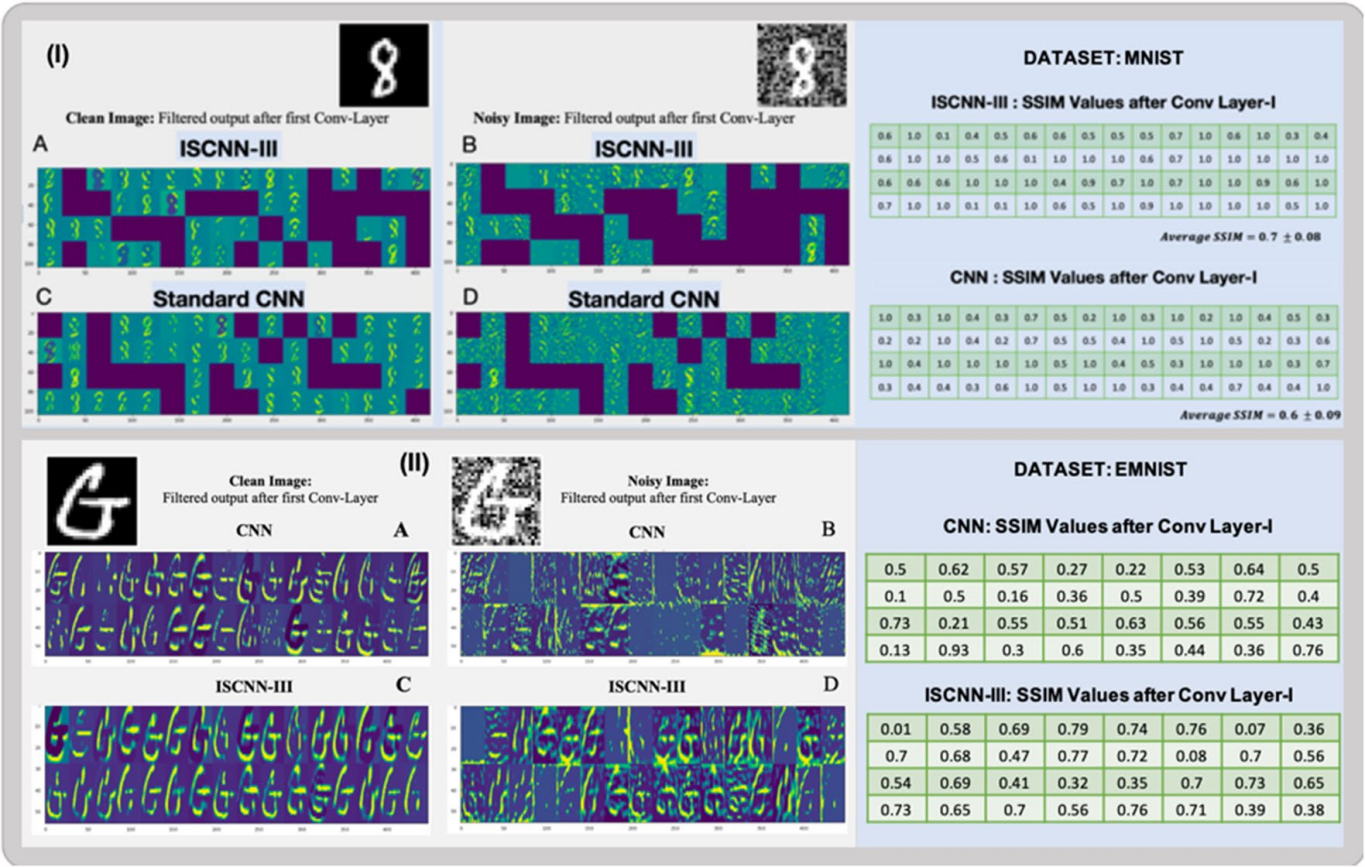
Filtered output after the first convolutional layer in the standard CNN and in ISCNN-III on both MNIST and EMNIST datasets. (**A** and **B**): The output of the filters to extract the high level features for clean and noisy digits. (**C** and **D**): The output of the filters to extract the high level features of clean and noisy letters.

The key benefit of introducing the Infor-Layer and Infor-Pool layer is that Inspite of noise present in the input signal the information layer helps to boost the different high level features corresponding to the CNN layers. The Structural Similarity Index (SSIM) helps to validate the above statement. SSIM measures the perceptual difference between two similar images. SSIM value + 1 indicates that the two given images are very similar while a value of 0 indicates the two given images are very different. The Fig. 2 shows the filtered output after the first convolutional layer in the standard CNN and in ISCNN-III for the standard MNIST and EMNIST datasets. These are the trained architectures with clean images only. The figure also presents the SSIM values calculated for the high level features of clean image with respect to the corresponding high level features of the noisy images for standard CNN and ISCNN-III. The standard CNN yielded a Structural Similarity Index (SSIM) of 0.6 ± 0.09 for the MNIST datasets, while the ISCNN-III model produced a higher SSIM of 0.7 ± 0.08. For the EMNIST datasets, the standard CNN recorded an SSIM of 0.4 ± 0.19, and the ISCNN-III model achieved again a higher SSIM of 0.5 ± 0.2. This shows that the information layer introduced in standard CNN helps to boost the high level features. Figure 2 emphasise only on the first layer of CNN but when observed on whole architecture the information layer/or polling introduced at every convolutional layer will enhance the different level of features at the corresponding layers and enhance the performance. The filtered output after each layer of standard CNN and ISCNN-III is shown in Figure S4 and Figure S5.

#### Comparative evaluation of ISCNN

Three variants of ISCNN, namely ISCNN-I, ISCNN-II, and ISCNN-III are considered, whose architectures are specified in Table 1 and in Table S2 for MNIST and EMNIST dataset respectively . In ISCNN-I, the Max-Pool layers of conventional CNN are replaced with the Infor-Layers, whereas; In ISCNN-II, the Infor-Layer is introduced at a single place only with an exponential membership function; and ISCNN-III uses both the Infor-Layer and Max-Pool, and combines the sigmoid and exponential gain membership functions. Table 2 summarizes the comparison of ISCNN with conventional CNN in terms of varying PSNR for the MNIST dataset. The highest performance is achieved by ISCNN-III. Table 3 presents the comparison between ISCNN-III and CNN on the EMNIST dataset. It is evident from the results that, at a lower PSNR range, the introduction of Infor-layer/Infor-pool significantly improves the performance of conventional CNN.

**Table 1:** Experimental designs (MNIST): Infor-set based convolutional neural networks.

Layers	CNN	ISCNN-I	ISCNN-II	ISCNN-III
Layer-1			Infor-Laye (Exp)	Infor-Layer (Sig-Exp)
Layer-2	Conv (Relu)	Conv (Relu)	Conv (Relu)	Conv (Relu)
Layer-3	Max Pool	Infor-Pool (Sig)	Max Pool	Infor-Pool (Sig)
Layer-4	Conv (Relu)	Conv (Relu)	Conv (Relu)	Conv (Relu)
Layer-5	Max Pool	Infor-Pool (Sig)	Max Pool	Infor-Pool (Sig)
Layer-6	Conv (Relu)	Conv (Relu)	Conv (Relu)	Conv (Relu)
Layer-7	Flatten	Flatten	Flatten	Flatten
Layer-8	Dense-softmax	Dense-softmax	Dense-softmax	Dense-softmax

**Table 2:** Experimental results (MNIST): Infor-set based convolutional neural networks.

PSNR	CNN (%)	ISCNN-I (%)	ISCNN-II (%)	ISCNN-III (%)
$\infty$	**99.30**	**99.30**	**99.30**	**99.30**
11.20	98.70	98.40	99.10	**99.20**
09.60	97.40	97.70	**98.90**	**98.90**
08.58	92.80	96.50	98.60	**98.80**
07.26	81.80	94.30	**98.00**	**98.00**
06.44	65.00	90.60	96.20	**96.40**

Significant values are in bold.

**Table 3:** Experimental results (EMNIST): Infor-Set Based Convolutional Neural Networks.

PSNR	CNN (%)	ISCNN-I (%)	ISCNN-II (%)	ISCNN-III (%)
$\infty$	87.8	86.8	86.4	87.0
12.06	70.1	71.4	71.7	76.9
10.49	53.3	61.5	63.9	69.6
09.07	36.1	49.0	54.5	58.3
07.79	23.6	36.2	44.6	45.3
07.14	15.8	25.4	32.8	33.4

Additional metrics including precision, recall, F1, and ROC-AUC score are also computed to analyse and compare ISCNN-III with traditional CNN at PSNR = 6.11 as shown in Tables 4 and 5 for MNIST and EMNIST datasets respectively. The layer details of the ISCNN architectures are shown in Table 6 for MNIST dataset. The layer details for EMNIST dataset are shown in Table S3.

**Table 4:** Experimental results (MNIST): performance comparison of ISCNN-III with CNN.

Digits	ISCNN-III	CNN	ISCNN-III	CNN	ISCNN-III	CNN
	Precision		Recall		F1-score	
0	0.98	0.92	0.99	0.95	0.98	0.94
1	0.99	0.98	0.81	0.44	0.89	0.6
2	0.95	0.78	0.91	0.79	0.93	0.79
3	0.98	0.98	0.93	0.71	0.96	0.83
4	0.85	0.87	0.98	0.77	0.91	0.81
5	0.94	0.8	0.95	0.76	0.95	0.78
6	0.99	0.96	0.95	0.78	0.97	0.86
7	0.99	0.93	0.9	0.71	0.94	0.8
8	0.74	0.34	0.98	0.98	0.85	0.5
9	0.96	0.93	0.90	0.58	0.93	0.72
ROC AUC SCORE (ISCNN-III)					0.99	
ROC AUC SCORE (CNN)					0.98

**Table 5:** Experimental Results (EMNIST): Performance Comparison of ISCNN-III with CNN.

Letters/Digits	Precision		Recall		F1-score	
	ISCNN-III	CNN	ISCNN-III	CNN	ISCNN-III	CNN
'0'	0.6	0.44	0.13	0.01	0.21	0.02
'1'	0.5	0	0	0	0	0
**'2'**	0.98	0.87	0.39	0.21	0.56	0.34
'3'	0.92	0.98	0.74	0.2	0.82	0.33
'4'	0.81	0.83	0.3	0.01	0.44	0.02
'5'	0.7	0.74	0.52	0.08	0.6	0.14
'6'	1	0	0.05	0	0.1	0
'7'	0.82	1	0.19	0	0.31	0
'8'	0.29	0.19	0.65	0.69	0.4	0.3
'9'	0.5	0	0.07	0	0.13	0
'A'	0.33	0.43	0.68	0.03	0.44	0.05
'B'	0.17	0.05	0.94	0.94	0.29	0.1
'C'	0.9	0	0.22	0	0.35	0
'D'	0.85	0.46	0.2	0.04	0.32	0.08
'E'	0.55	0.34	0.91	0.78	0.68	0.47
'F'	0.24	0.71	0.29	0.03	0.26	0.05
'G'	0.3	0.19	0.82	0.76	0.44	0.31
'H'	0.9	0.88	0.11	0.02	0.2	0.03
'I'	0.7	0	0.04	0	0.07	0
'J'	0.83	1	0.12	0	0.22	0
'K'	0.76	0.62	0.19	0.01	0.31	0.02
'L'	0.25	0	0	0	0	0
'M'	0.92	0.89	0.55	0.17	0.68	0.28
'N'	0.92	0	0.09	0	0.16	0
'O'	0.56	0	0.14	0	0.23	0
'P'	0.54	0.28	0.23	0.03	0.32	0.05
'Q'	0.21	0.22	0.87	0.74	0.34	0.34
'R'	0.27	0.25	0.53	0.21	0.36	0.23
'S'	0.85	0.87	0.41	0.03	0.55	0.06
'T'	1	0	0.04	0	0.08	0
'U'	0.93	1	0.07	0.01	0.13	0.01
'V'	0.9	0	0.07	0	0.12	0
'W'	0.84	0.91	0.46	0.03	0.6	0.05
'X'	0.91	0.89	0.21	0.02	0.35	0.04
'Y'	0.61	1	0.08	0	0.14	0
'Z'	0.44	0.41	0.83	0.75	0.57	0.53
'a'	0.26	0.26	0.41	0.08	0.32	0.12
'b'	0.55	0.03	0.04	0.01	0.07	0.01
'd'	0.79	0.53	0.27	0.02	0.4	0.04
'e'	0.84	0.7	0.6	0.3	0.7	0.42
'f'	0.27	0.44	0.69	0.07	0.39	0.12
'g'	0.17	0.09	0.65	0.64	0.27	0.16
'h'	1	0.5	0.01	0	0.02	0
'n'	0.87	0	0.03	0	0.06	0
'q'	0.08	0.12	0.56	0.47	0.14	0.19
'r'	0.85	0	0.09	0	0.16	0
't'	0.32	0	0.08	0	0.13	0
ROC AUC SCORE (ISCNN-III) = 0.95						
ROC AUC SCORE (CNN) = 0.86

**Table 6:** Layer details of CNN and ISCNN models.

Conventional CNN		ISCNN-I		ISCNN-II		ISCNN-III	
Layer	Output Shape	Layer	Output Shape	Layer	Output Shape	Layer	Output Shape
Input Layer	[(None, 28, 28, 1)]	Input Layer	[(None, 28, 28, 1)]	Input Layer	[(None, 28, 28, 1)]	Input Layer	[(None, 28, 28, 1)]
Conv-Layer (2D)	[(None, 26, 26, 64)]	Conv-Layer (2D)	[(None, 26, 26, 64)]	Infor-Layer (Exp)	[(None, 28, 28, 1)]	Infor-Layer (Sig-Exp)	[(None, 28, 28, 1)]
Max-Pool (2D)	[(None, 13, 13, 64)]	Infor-Pool (Sig)	[(None, 13, 13, 64)]	Conv-Layer (2D)	[(None, 26, 26, 64)]	Conv-Layer (2D)	[(None, 26, 26, 64)]
Conv-Layer (2D)	[(None, 11, 11, 64)]	Conv-Layer (2D)	[(None, 11, 11, 64)]	Max-Pool (2D)	[(None, 13, 13, 64)]	Infor-Pool (Sig)	[(None, 13, 13, 64)]
Max-Pool (2D)	[(None, 5, 5, 64)]	Infor-Pool (Sig)	[(None, 5, 5, 64)]	Conv-Layer (2D)	[(None, 11, 11, 64)]	Conv-Layer (2D)	[(None, 11, 11, 64)]
Conv-Layer (2D)	[(None, 3, 3, 64)]	Conv-Layer (2D)	[(None, 3, 3, 64)]	Max-Pool (2D)	[(None, 5, 5, 64)]	Infor-Pool (Sig)	[(None, 5, 5, 64)]
Flatten	(None, 576)	Flatten	(None, 576)	Conv-Layer (2D)	[(None, 3, 3, 64)]	Conv-Layer (2D)	[(None, 3, 3, 64)]
Dropout	(None, 576)	Dropout	(None, 576)	Flatten	(None, 576)	Flatten	(None, 576)
dense_1	(None, 100)	dense_1	(None, 100)	Dropout	(None, 576)	Dropout	(None, 576)
dense_2	(None, 10)	dense_2	(None, 10)	dense_1	(None, 100)	dense_1	(None, 100)
				dense_2	(None, 10)	dense_2	(None, 10)

**Figure 3:**
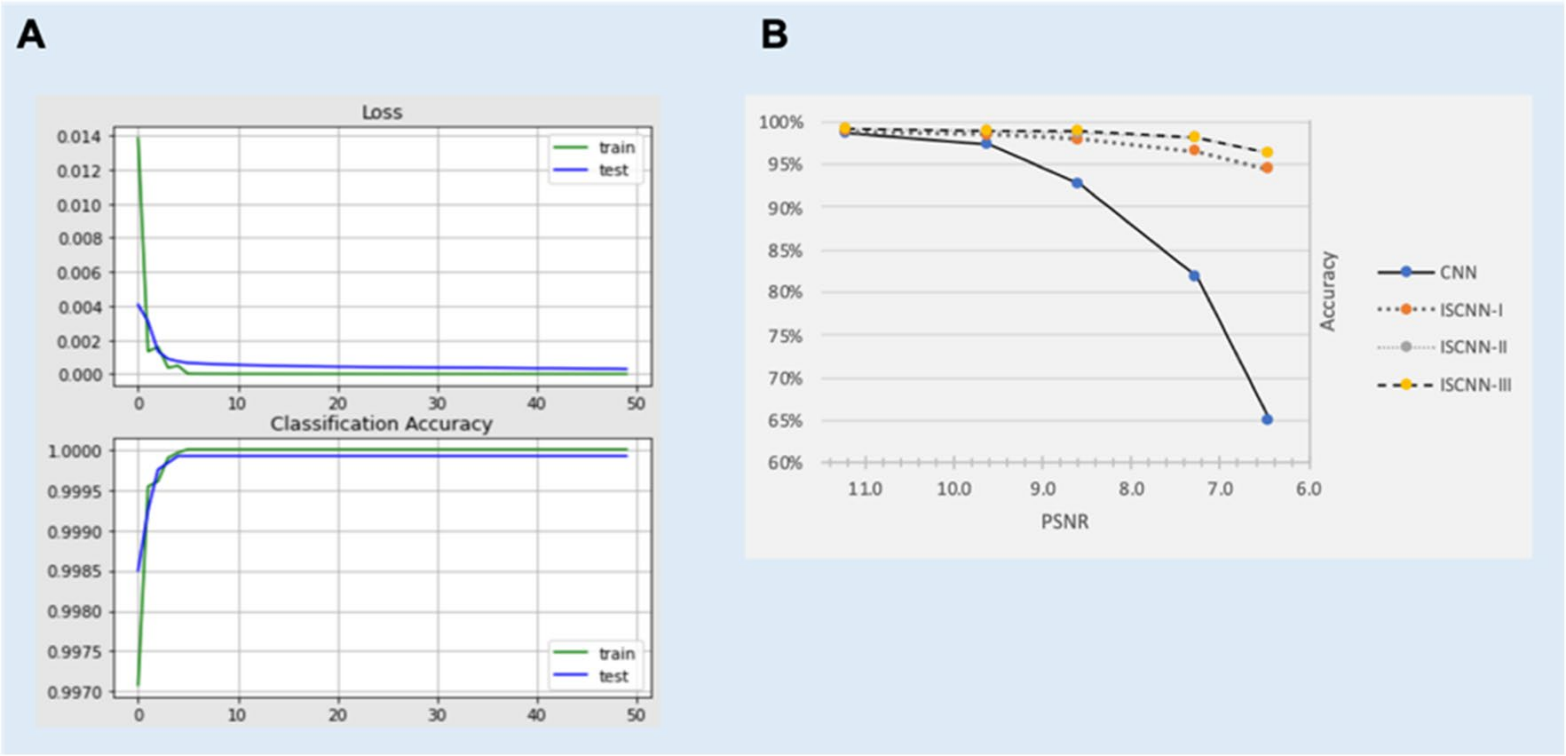
(**A**) The training loss and classification accuracy of ISCNN-III (**B**) Comparison of CNN, ISCNN-I, ISCNN-II, and ISCNN-III with varying PSNR on the MNIST dataset. The proposed models show noise robustness while the performance of CNN decreases sharply.

The model is evaluated using fivefold cross-validation. The test set for MNIST has 12,000 samples, which is nearly the same size as the training dataset which is having 10,000 samples. Before splitting, the training dataset is shuffled. The ISCNN-III (the best model) architecture's learning accuracy and loss are shown in Fig. 3A.

While the performance of all ISCNN versions is comparable to that of classic CNN models when the data is clean, it is significantly better than traditional CNN when the test dataset has much more noise. Figure 3B depicts a comparison between CNN and other ISCNN variants with increasing PSNR.

**Figure 4:**
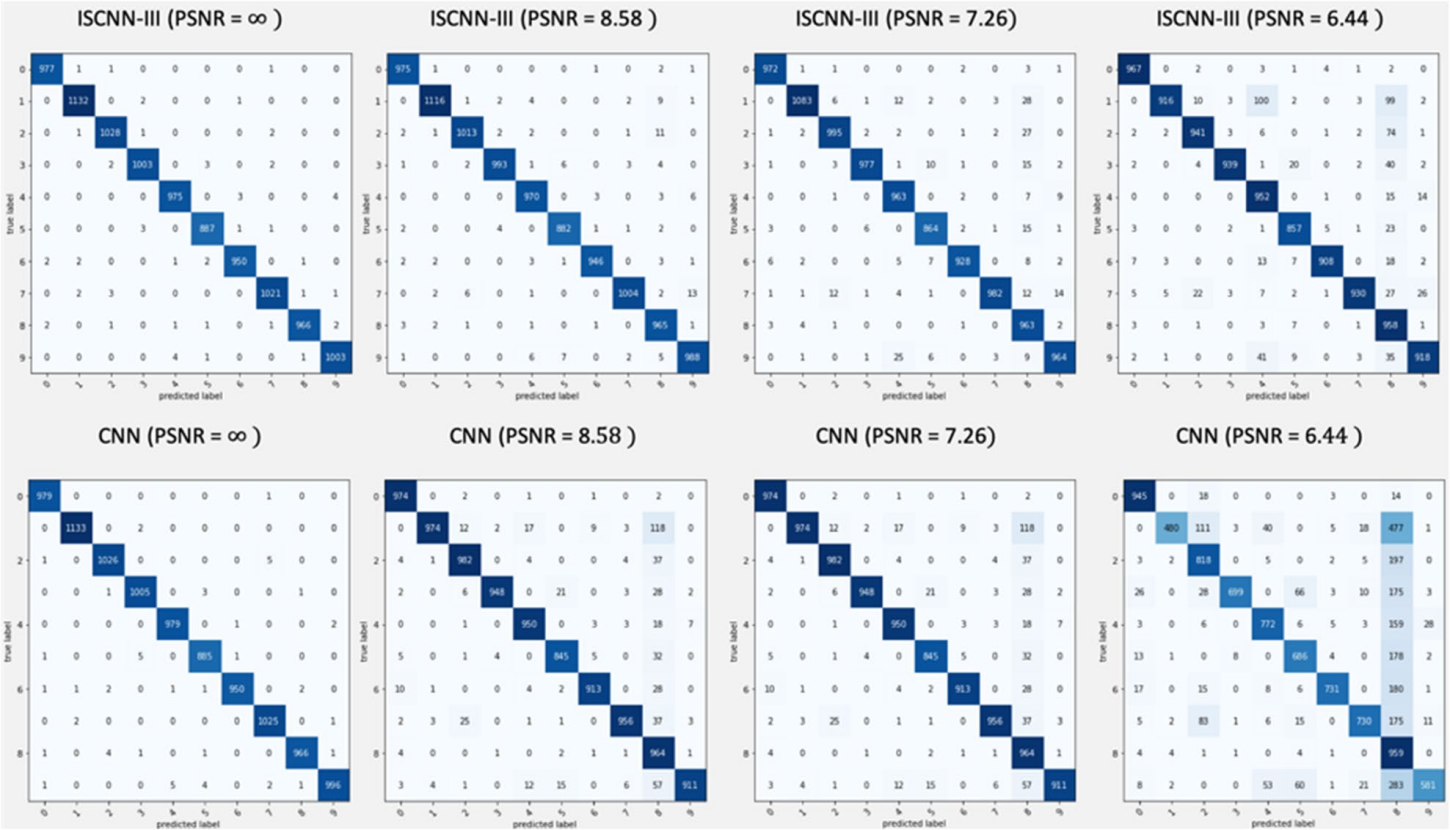
Confusion matrices for ISCNN-III and CNN for varying PSNR values for MNIST dataset.

Figure 4 illustrates the confusion matrices for MNIST dataset comparing CNN and ISCNN-III performance with varying PSNR. Figure S6 shows the same comparison for the EMNIST dataset. When images are not contaminated by noise, the performance of classical CNN and information set theory-based CNN is almost similar. However, the performance of classical CNN drastically declines with decreasing values of PSNR. A one-digit sample with two different PSNR values is shown in Figure S1. A number of wrongly classified and correctly classified samples are included in Figure S2 and Figure S3, respectively.

### Infor-set based deep feedforward networks (ISNN)

**Figure 5:**
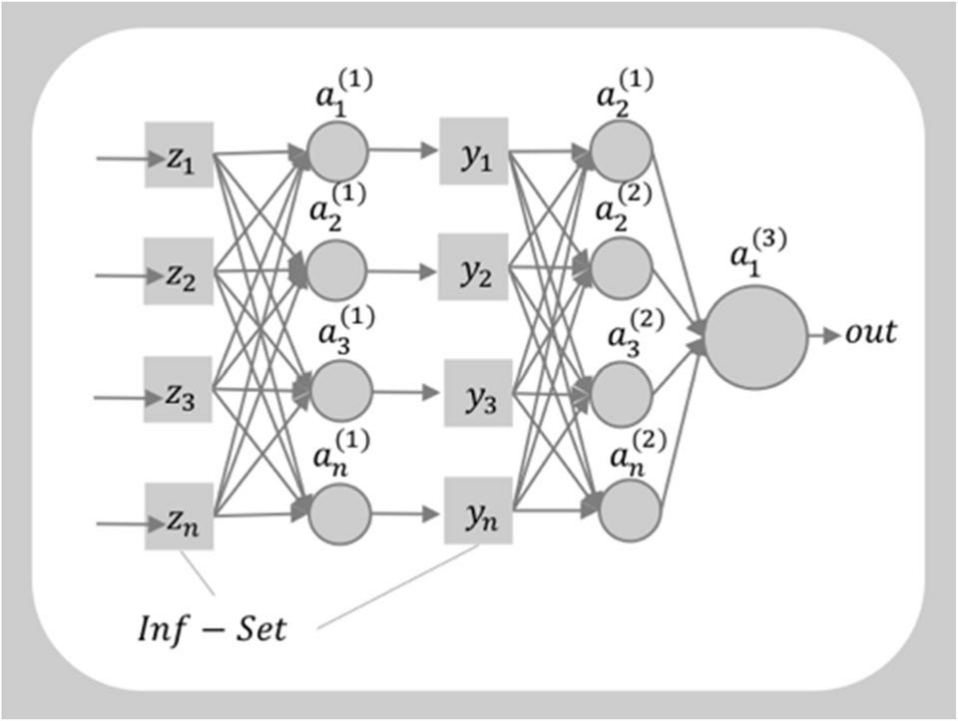
Infor-Set based Deep Feedforward Network: Infor-Layer is introduced in between all the layers of DL-NN.

The architecture of ISNN consisting of nodes (circles) and the connections (lines) between the nodes is shown in Fig. 5. The deep neural networks have multiple hidden layers to create deeper representations on each layer. Only two hidden layers have been shown for simplicity and ease of demonstration. The output from the node on the left is connected to the node on the right through a matrix multiplication in between weights and input, which is further passed through an appropriate nonlinear activation function. The output of neuron after each layer is calculated as:

2 $$a_{j}^{\left( k \right)} = f\left( {\mathop \sum \limits_{{\begin{array}{*{20}c} {i = 1} \\ {j = 1} \\ \end{array} }}^{n} w_{ji} x_{i} + b} \right)$$

where superscript $(k)$ denotes the layer number, ${x}_{i}=({x}_{1},{x}_{2}\dots , {x}_{n})$ is the input feature, ${w}_{ji}$ is the weight connected from ${i}{th}$ to ${j}{th}$ nodes, and $f$ is a nonlinear activation function.

In ISNN, Infor-Layer is applied after source and after every dense layer. The output is the product of information source values with the corresponding membership function values, as explained earlier. For example, the information set value ${z}_{i}$ is calculated as

3 $$z_{i} = x_{i} G_{s} \left( {X_{i} } \right)$$

where, ${G}_{s}\left({X}_{i}\right)$ is the suitable membership function such as sigmoid, exponential, gaussian, etc. Similarly, *Infor-Layer* is applied after each layer.

#### Evaluation of infor-set deep feedforward networks (ISNN)

The comparison of the architecture and the results with DL-NN and ISNN is summarized in Tables 7 and 8, respectively. Here, the performance metric of "accuracy" is used to measure how well the model predicts the test data set from both positive and negative classes (Supplementary Information). The 'relu' activation function is used for initial dense layers, and the 'Softmax' activation function for the final layer. The 'sigmoid' is used as a membership function for Infor-Set calculation. With a batch size of 32, training is done over five epochs. Out of six experiments, the best outcomes for each were taken into consideration.

**Table 7:** Architecture comparison between DL-NN and ISNN for MNIST dataset.

LAYERS	DL-NN	ISNN
Layer-1	-	Infor-Layer
Layer-2	Dense-relu	Dense-relu
Layer-3	-	Infor-Layer
Layer-4	Dense-relu	Dense-relu
Layer-5	Dense-softmax	Dense-softmax

**Table 8:** Performance comparison between DL-NN and ISNN based on different PSNR for MNIST dataset.

PSNR	DL-NN (%)	ISNN (%)
**Inf**	**98.05**	**97.09**
11.20	70.05	**88.44**
09.60	62.18	**82.49**
08.58	53.23	**75.45**
07.26	44.74	**66.17**
06.44	36.72	**56.17**

Significant values are in bold.

#### Description of layer-I and layer-III in Table 7

In ISNN, two Infor-Layers are introduced which do not exist in DL-NN. In layer-1, instead of linear activation function, the Infor-Layer is considered, whereas layer III encapsulates the information from layer -II's output.

In Table 8, for the uncorrupted data (PSNR = Inf) the performance of both the architectures for MNIST dataset is identical. Afterward, with the introduction of noise (with varying PSNR values) the performance of conventional networks deteriorated sharply, whereas ISNN exhibited robustness to noise. It is clear from the table that the ISNN can also maintain high accuracy of 56.17% compared to 36.77% of DL-NN when the input image is corrupted with high noise (PSNR = 6.4: as minimal information of actual image due to noise).

It is evident from Table 9 that even at PSNR = 6.44, the standard architectures DL-NN and CNN yields 36.72% and 65%, respectively. One of the proposed variants yields a significantly better performance of 96.40%, which is almost consistent even at the high noise level.

**Table 9:** Comparison of different models on noisy MNIST data.

PSNR	DL-NN (%)	ISNN (%)	CNN (%)	ISCNN-III (%)
11.20	70.05	88.44	98.70	99.20
09.60	62.18	82.49	97.40	98.90
08.58	53.23	75.45	92.80	98.80
07.26	44.74	66.17	81.80	98.00
06.44	36.72	56.17	65.00	96.40

## Discussion

In the present work, the capability of Infor-Set theory to handle uncertainty is exploited by integrating it with Deep Learning architectures to extract and enhance the actual information buried under the noise. The proposed architecture's efficacy is showcased using the MNIST database of handwritten digits and the EMNIST Balanced dataset, which is an extensive and inclusive compilation of handwritten characters and digits. To validate the proposed approach's noise tolerance, both datasets are degraded with varying levels of Signal-to-Noise Ratio (SNR).

The approach is general and can be easily applied to many deep networks. In the present work, the proposed technique is used to develop one variant of DL-NN and three variants of CNN. In ISNN (a variant of DL-NN), an Infor-Layer is introduced after the input layer and between the standard layers of DL-NN. The three variants of ISCNN are ISCNN-I to ISCNN-III. In ISCNN-I, Infor-Pool layer is introduced to replace the Max-Pool layer in CNN. In this architecture, the localized information is extracted in terms of pooling for the features extracted by filters of CNN. In Max-pool, the maximum value of the window is taken, but it may provide false information due to corrupted input. Whereas, in ISCNN-II, the Infor-Pool layer is introduced just after the input layer. The filters in CNN architecture are used for feature engineering, but if the source is corrupted, the features are not efficient. In ISCNN-III, both the Infor-Pool in place of Max-Pool and Infor-Layer after the input layer is introduced so that the raw data is not directly inserting into the Conv-Layer due to this. The effective information is extracted, which improves the quality of features extracted using the filters of Conv-Layer.

These all architectures are tested with different gain functions and their combinations. In the present work only the selected membership functions are used; however, a dynamic membership function can be devised that can adapt to the environment. For the uncorrupted data, the performance of proposed architectures was identical to conventional architectures; however, there is a significant improvement and consistency in performance on the degraded MNIST and EMNIST data at different levels of PSNR.

It's worth noting that the proposed technique isn't a denoising technique. Instead, it can improve the useful information, which in turn improves noise handling. This does not necessarily imply that the proposed models are denoising; nonetheless, the noise may be reduced as a result of the procedure. The proposed method is less computationally expensive and capable of boosting effective information, which improves noise handling.

One limitation of the proposed method is that Infor-set layers do not contain trainable parameters. Therefore, all of ANN's layers can’t be replaced with Infor-set layers; otherwise, there would be no room for learning. In the future, the Generalised Entropy Function (GEF)^12^ will be tested which has the potential to replace the layers of CNN since its parameters not only collect information from the source but can also be trained to capture the relationship between input and output. Furthermore, compared to conventional architectures, the number of parameters that must be learned will be substantially reduced.

Because noise/irrelevant features in the proposed work may execute self-suppression or self-consolation, theoretical insights from combining information set theory with the activation function can be investigated to alleviate overfitting concerns. In the future, this strategy will be applied to broader real-world problems, such as medical image segmentation. The proposed techniques can also be extended for other deep learning architectures such as Autoencoders, U-Net, and RNN, to name a few.

## Methods

### Information set theory

Fuzzy set theory was proposed to deal with uncertainty present in the crisp sets (conventional sets) by characterizing the imprecise, vague, or missing information. The fuzzy sets are formulated as a pair (*member, membership grade*), where the membership grade is defined in the interval [0,1] and captures the belongingness of the member in a fuzzy set.

The Information set (Infor-set) theory employs an entropy framework to transform the fuzzy set into information values and create the information set. The aggregation of information values imparts the overall uncertainty.

#### Conversion of a fuzzy set into an information set

Consider an attribute $X$ with the following set of values:

4 $${X=\{x}_{1}, {x}_{2}\dots ,{x}_{n}\}$$

The set of values that ${x}_{i}$ indicates is denoted as

5 $${I}_{\rm X}=\left\{{I}_{\rm X}\left({x}_{i}\right)\right\} \forall {x}_{i}\in X$$

where $${I}_{\rm X}\left({x}_{i}\right)$$ is the individual information source value and $${I}_{\rm X}$$ is the collection of information source values from $$X$$ . To create fuzzy sets, these values are segregated into *K* soft classes. The *k*th fuzzy set ($${F}_{X}^{k})$$ is represented as the pair $${(I}_{\rm X}\left({x}_{i}\right), {\mu }_{X}^{k} \left({x}_{i}\right))$$, where $${I}_{\rm X}\left({x}_{i}\right)\mathrm{ and }{\mu }_{X}^{k} \left({x}_{i}\right)$$ are the information source values and the corresponding membership grades, respectively.

To measure the uncertainty in fuzzy sets, the conventional entropy functions such as Shannon and Pal and Pal^5^ are not suitable as they provide the uncertainty in a probability but not possibility domain. Typically, for fuzzy sets, various fuzzy entropy functions are used. However, the limitation of these functions is that they can only capture the uncertainty in membership grades and not in information source values. As a remedy, the generalized Hanman-Anirban entropy function has been developed^8^, which combines the information source values and the associated gain as a single entity termed as “information value”.

For the fuzzy set ${F}_{X}^{k}$, the uncertainty, or information in ${I}_{\rm X}$ is formulated as:

6 $${E}_{X}^{k}=\sum_{i}{I}_{X}\left({x}_{i}\right){g}_{X}^{k}\left({x}_{i}\right)$$

with

$${g}_{X}^{k}\left({x}_{i}\right)={e}^{-{({a}_{X}^{k}{{(I}_{X}\left({x}_{i}\right))}^{3}+{b}_{X}^{k}{{(I}_{X}\left({x}_{i}\right))}^{2}+{c}_{X}^{k}{(I}_{X}\left({x}_{i}\right))+{d}_{X}^{k})}^{{\alpha }_{X}^{k}}}$$

where ${g}_{X}^{k}$ is the gain function representing the uncertainty in the information source value. The uncertainty is further converted into the information values (entropy values) in (6). While the gain function is data-dependent, Infor-set theory provides the flexibility to choose different membership functions (Gaussian, Sigmoid, etc.) by optimizing ${a}_{X}^{k}, {b}_{X}^{k}, {c}_{X}^{k}, and {d}_{X}^{k}$ belonging to the *k*th fuzzy set. All the information values are collected using a generalized Hanman-Anirban entropy function to form the *Information set*,

7 $${S}_{X}^{k}=\left\{{I}_{X}\left({x}_{i}\right){g}_{X}^{k}\left({x}_{i}\right)\right\} \forall {x}_{i}\in X$$

and the sum of all the information values in ${S}_{X}^{k}$ is the effective information ${E}_{X}^{k}$. The normalized effective information ${E}_{XN}^{k}$ is obtained as

8 $${E}_{XN}^{k}=\frac{1}{\left|X\right|}\sum_{i}{I}_{X}\left({x}_{i}\right){g}_{X}^{k}\left({x}_{i}\right)$$

The definitions related to Information source values, Information set, and for the normalized information are discussed in^3^ in more detail.

#### Procedure to compute the effective information using the Gaussian membership function

Consider a data matrix ${\varvec{X}}$ of size ${\varvec{d}}\times {\varvec{m}}$.

9 $$X = \left[ {\begin{array}{*{20}c} {\begin{array}{*{20}c} {x_{{11}} } \\ {\begin{array}{*{20}c} \vdots \\ {x_{{{\text{i}}1}} } \\ \vdots \\ \end{array} } \\ {x_{{{\text{d}}1}} } \\ \end{array} } & {\begin{array}{*{20}c} {\begin{array}{*{20}c} \cdots \\ {\begin{array}{*{20}c} \ddots \\ \cdots \\ \ddots \\ \end{array} } \\ \cdots \\ \end{array} } & {\begin{array}{*{20}c} {x_{{1{\text{j}}}} } \\ {\begin{array}{*{20}c} \vdots \\ {x_{{{\text{ij}}}} } \\ \vdots \\ \end{array} } \\ {x_{{{\text{dj}}}} } \\ \end{array} } & {\begin{array}{*{20}c} \cdots \\ {\begin{array}{*{20}c} \ddots \\ \cdots \\ \ddots \\ \end{array} } \\ \cdots \\ \end{array} } \\ \end{array} } & {\begin{array}{*{20}c} {x_{{1{\text{m}}}} } \\ {\begin{array}{*{20}c} \vdots \\ {x_{{{\text{im}}}} } \\ \vdots \\ \end{array} } \\ {x_{{{\text{dm}}}} } \\ \end{array} } \\ \end{array} } \right]_{{d \times m}}$$

- **Step 1** Computation of mean (${\mu }_{j}$) and variance $( {\sigma }_{j})$ of the jth attribute considering only one soft class per attribute.
  10 $$\mu_{j} = \frac{1}{d}\mathop \sum \limits_{i = 1}^{d} x_{ij} , \quad j = 1, \ldots ,m$$
  11 $$\sigma_{j} = \mathop \sum \limits_{i = 1}^{d} \left( {x_{ij} - \mu_{j} } \right)^{2} , \quad j = 1, \ldots ,m$$
  where, $${{\varvec{x}}}_{{\varvec{j}}}$$ is:
  $${{\varvec{x}}}_{{\varvec{j}}}=\left[\begin{array}{c}{x}_{1j}\\ \begin{array}{c}\vdots \\ {x}_{ij}\\ \begin{array}{c}\vdots \\ {x}_{dj}\end{array}\end{array}\end{array}\right], \quad j=1,\dots ,m$$
- **Step 2** Calculation of membership function value for each information source for the jth attribute.
  12 $${\text{G}}\left( {x_{ij} } \right) = e^{{ - \frac{1}{2}\left( {\frac{{x_{ij} - \mu_{j} }}{{\sigma_{j} }}} \right)^{2} }} , \quad j = 1, \ldots ,m$$
- **Step 3** Calculation of information values for the information source matrix ${\mathbb{X}}$.
  13 $$\begin{gathered} S_{X} \left( {x_{ij} } \right) = x_{ij} {\text{G}}\left( {x_{ij} } \right), \quad i = 1, \ldots ,{\text{d }} \hfill \\ \quad \quad \quad \quad \quad \quad \quad \quad \quad \quad j = 1, \ldots ,{\text{m}} \hfill \\ \end{gathered}$$
  where,
  $${S}_{X}=\left\{{S}_{X}\left({x}_{ij}\right)\right\}, \quad i=1,\dots ,\mathrm{d } \quad j=1,\dots ,\mathrm{m}$$
- **Step 4** Computation of effective information
  14 $$E_{i} = \frac{1}{\left| X \right|}\mathop \sum \limits_{{{\text{i}} = 1}}^{d} \left\{ {S_{X} \left( {x_{ij} } \right)} \right\}, \quad j = 1, \ldots ,{\text{m}}$$

For the noisy data with varying SNR, instead of trained generalized gain function, the sigmoid and exponential membership functions are used, which give comparable results in the experiments. These (sigmoid & exponential) are the standard functions and can be derived from the generalized gain function.

#### Connection between the information set and ‘swish’

The s*wish* activation function is represented as:

15 $$f\left( x \right) = x.sigmoid\left( {\beta x} \right)$$

Here the connection between the '*swish'* activation function and the information sets can be clearly observed. In fact, the above equation is a special case of Eq. (6) with gain function ${g}_{X}^{k}\left({x}_{i}\right)=$ $sigmoid(\beta x)$.

Typically, in Artificial Neural Network (ANN) the outcome of every neuron captures the information in the form of $\sum {x}_{i}{w}_{i}$, similar to what Infor-set theory is formulated in the form of $\sum_{i}{I}_{X}\left({x}_{i}\right){g}_{X}\left({x}_{i}\right)$. However, the two has two major differences. First, in ANN, the weight generation does not follow any standard distribution function; instead, training develops these; In contrast, the Infor-set theory acquire weights with the help of a generalized gain function. Second, weights in ANNs are determined by the input–output relationship. The Infor-set theory, on the other hand, does not extract any information based on this relationship; instead, it merely pulls information from input.

### Infor-layer

- **Step 1** Information Source Values
  The information source values are attributes/features of an image, which can be represented as
  16 $$I_{X} = \left\{ {I_{X} \left( {x_{ijk} } \right)} \right\},\quad i = 1,2,3; \;j = 1, \ldots ,d; \;k = 1, \ldots ,m$$
- **Step 2** Information gain
  The Information gain is calculated for each element of the information source in a window with the help a generalized membership function as shown in (4). The present work uses the commonly used membership function(s) (Sigmoid and exponential). Example: The gain value for the sigmoid Membership function is calculated as:
  17 $$G_{s} \left( {X_{ijk} } \right) = \frac{1}{{\left( {1 + \exp \left( { - x_{ijk} } \right) } \right)}}$$
- **Step 3** Information set
  The information set is obtained by multiplying information source values with the corresponding information gain
  18 $$I_{X}^{1} \left( {X_{ijk} } \right) = x_{ijk} G_{s} \left( {X_{ijk} } \right)$$

The extracted Infor-Sets are the output of the Infor-Layer.

The proposed operation is general and can be applied to any multi-dimensional data. The procedure is demonstrated in the following steps by taking an example of a 3D image.

### Infor-pool

**Figure 6:**
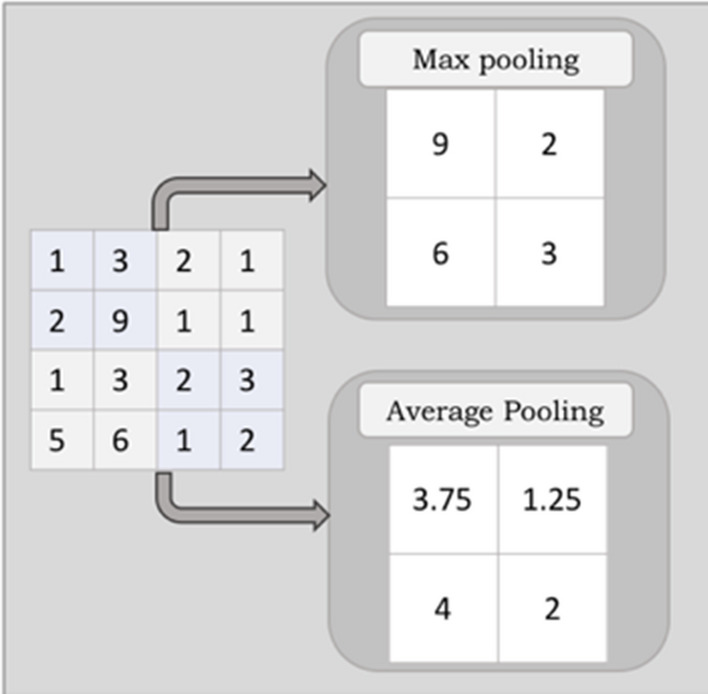
Illustration of Max Pooling and Average Pooling: In Max-Pool, the maximum value is chosen from the selected window, whereas Avg-pool determines the average value.

The function of the Pooling layer is to reduce the dimension of the feature map (number of pixels) by capturing the information contained in the region. This reduces the computational complexity of the network and, in turn, speed-up the operation. This operation involves no training of the parameters. However, it has hyperparameters, including the size of the filter, stride, and padding. The most common pooling layers are the Max pooling and the Average pooling. These operations with a stride of two and filter size 2 × 2 are shown in Fig. 6. Although these pooling operations provide exemplary performance in terms of positional invariance and reduce the size and complexity, there is significant information loss.

According to our literature review, little work has been done to address information loss in pooling operations. The maximum value is chosen from the feature map's window in the Max-pooling operation. In this operation, the most prominent value is chosen, while the other values are discarded. On average pooling, the average of the values in a window is selected.

**Figure 7:**
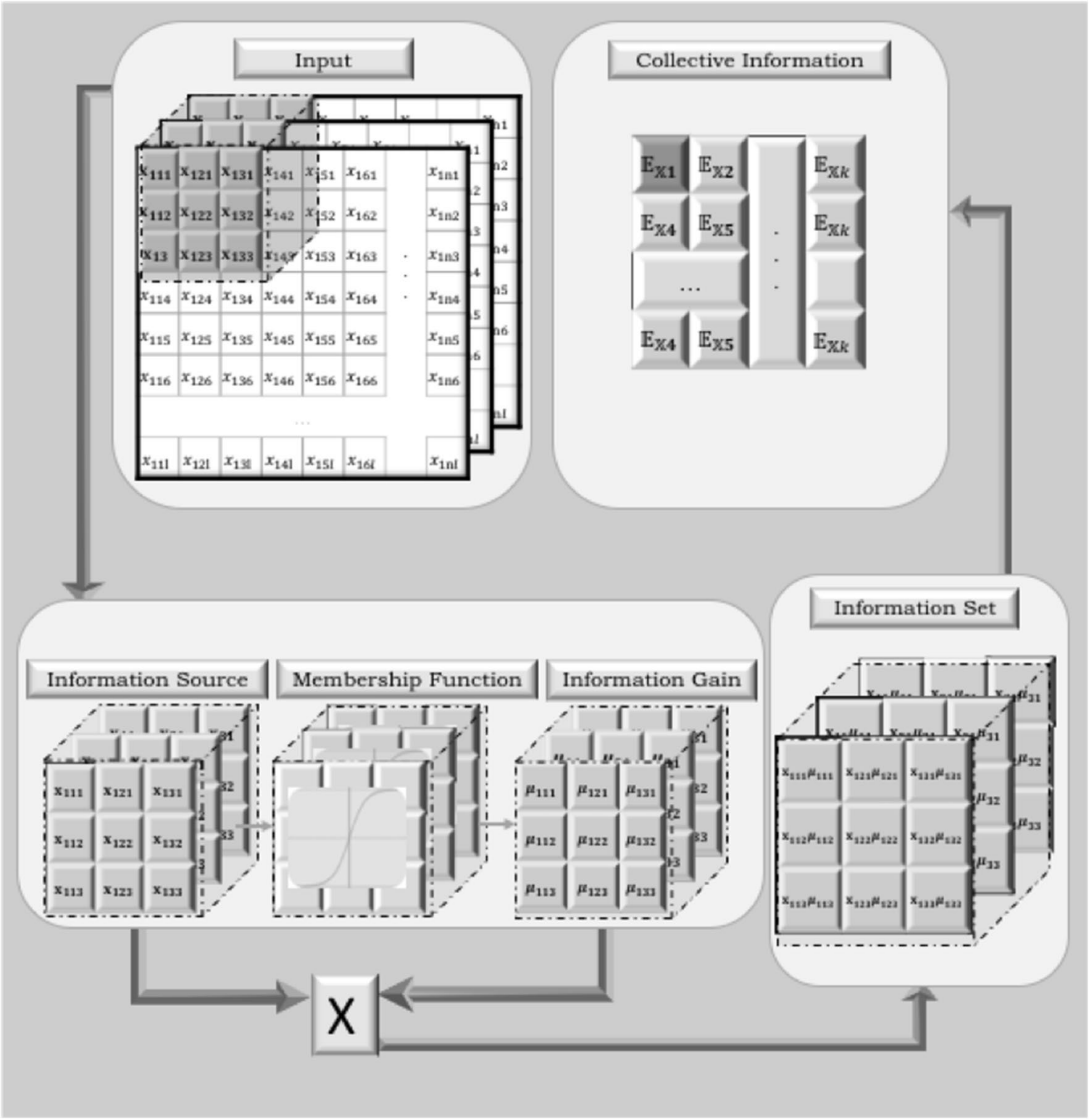
Infor-Set Based Pooling Layer: Steps for obtaining the effective/collective information are explained. First, the Information set is obtained by taking the product of the Information source and Information Gain, then the average value of this Information set is calculated to obtain the collective information. Afterward, the window/volume is shifted as per the chosen stride and padding.

The Infor-set based pooling captures the holistic information of a specific window weighted as per the input. Figure 7 shows the block diagram of the process.

- **Step 1** Information extraction
  Before information extraction, choose a non-overlapping window of size $m\times m \times m$ extracted from a input matrix of size $n\times n\times n$, which is obtained after the convolution operation (where $m<n$). In the figure a $3\times 3\times 3$ window is selected. Afterward, the information from the non-overlapping window is extracted by following the procedure explained in section (Infor-Layer).
- **Step-2** Collective information calculation
  After information extraction, the collective information contained in the selected window is captured with the help of the following equation:
  19 $$E_{Xi} = \frac{1}{M}\mathop \sum \limits_{j = 1}^{m} I_{X}^{1} \left( {X_{ijk} } \right)$$

To calculate the overall information from the entire input matrix, the window is shifted according to the chosen stride $\mathrm{^{\prime}}k\mathrm{^{\prime}}$ and calculate the next value of collective information by following the procedure as explained. After following the above steps, a new matrix of size S (3 × h × v) is obtained. The values of 'h' and 'v' are calculated as:

20 $$h = \frac{n + 2p - m}{k} + 1$$

21 $$v = \frac{n + 2p - m}{k} + 1$$

where $\mathrm{^{\prime}}n\mathrm{^{\prime}}$ is the size of the input matrix, $\mathrm{^{\prime}}p\mathrm{^{\prime}}$ is the applied padding, $\mathrm{^{\prime}}m\mathrm{^{\prime}}$ is the size of the window, and $\mathrm{^{\prime}}k\mathrm{^{\prime}}$ is the applied stride. The output matrix obtained after the Infor-Set-based pooling operation will be used as an input for future layers.

The proposed integration can enhance the effective information, which handles the noise better and may be suppressed in the process. This situation can be analysed by taking the following simple example:

Take an image and suppose it is corrupted by adding a salt and pepper noise. Afterward, take a small window for localized information and let this window have more black pixels than white before introducing noise. Then as per the steps of the Infor-set theory, let us derive the Gaussian MFs through the generalized information gain formalism, which as per the theory, gives a measure of uncertainty in the information source values. Afterward, an information set is prepared, which is a collection of the information values corresponding to the original source values, computed using the Hanman–Anirban entropy function. In this situation, if salt falls on any pixel, then according to Infor-set theory, see the belongingness of this particular salt concerning its neighborhood, this is very insignificant because it is dominated by the dark pixels. When salt noise is introduced, the source value of that specific pixel becomes higher, but its belongingness is very low due to the neighborhood. When the effective information is calculated by multiplying the information source with its belongingness, it reduces the effect of salt. On the other hand, in the case of pepper noise means the dark pixel is there. However, its belongingness is high, so if it is multiplied then, it still becomes dark. So, in this particular case, the Infor-set theory is trying to extract the actual information up to some extent and suppress the noise. Table S1 explains the significant differences between the proposed models and the most relevant conventional DL models in design and functionality.

## Supplementary Information

Supplementary Information.

## Data Availability

In this study, the datasets analyzed can be found in the MNIST Database of handwritten digits repository, which can be accessed via the URL http://yann.lecun.com/exdb/mnist/. The EMNIST dataset, on the other hand, can be obtained from the Kaggle website at https://www.kaggle.com/datasets/crawford/emnist.
