# Supplementary material for: Information set supported deep learning architectures for improving noisy image classification
Source: Sci Rep. 2023 Mar 17;13:4417. doi: 10.1038/s41598-023-31462-6 (PMC10023670; doi:10.1038/s41598-023-31462-6)
Supplement: Supplementary file 1 — Supplementary Information. [file 41598_2023_31462_MOESM1_ESM.docx]

Supplementary Information

**Information Set Supported Deep Learning Architectures for Improving Noisy Image Classification**

Saurabh Bhardwaj^1, *^, Yizhi Wang^2^, Guoqiang Yu^2^, and Yue Wang^2^

^1^Department of Electrical and Instrumentation Engineering, Thapar Institute of Engineering and Technology, Patiala, Punjab 147004, India

^2^Department of Electrical and Computer Engineering, Virginia Polytechnic Institute and State University, Arlington, VA 22203, USA

**Supplementary Figures**

**
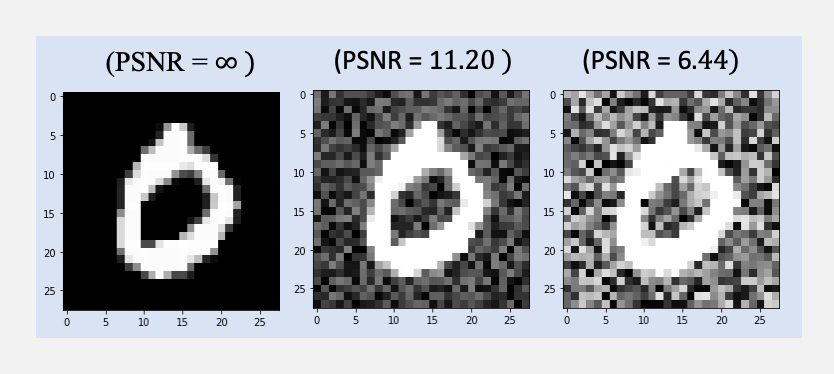
**

**Figure S1.** One handwritten digit samples with varying PSNR values

**
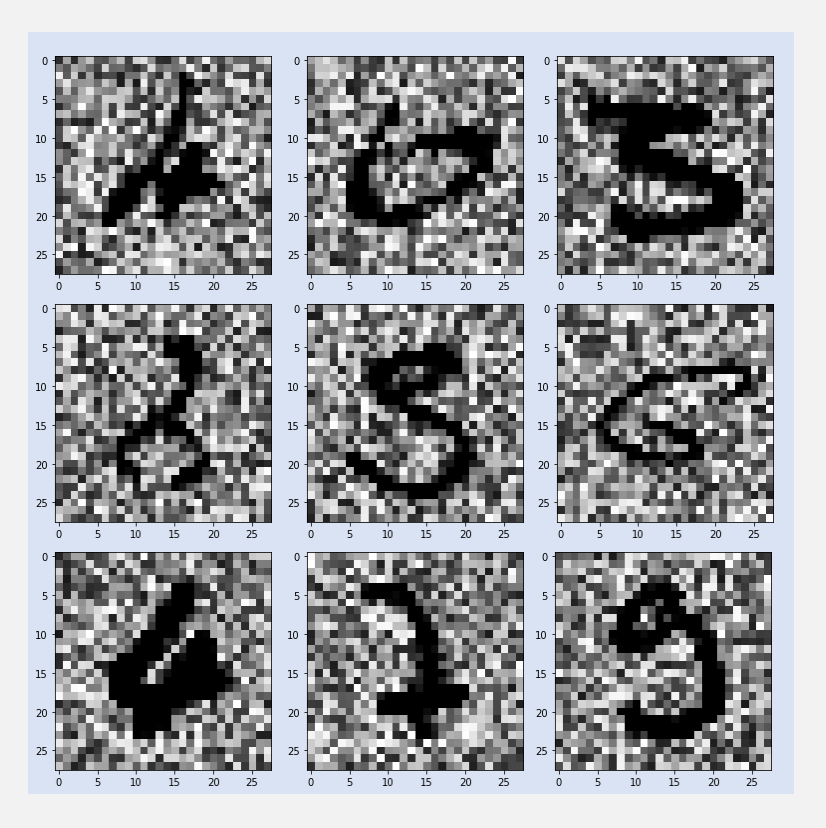
**

**Figure S2.** Samples with incorrect classification

**
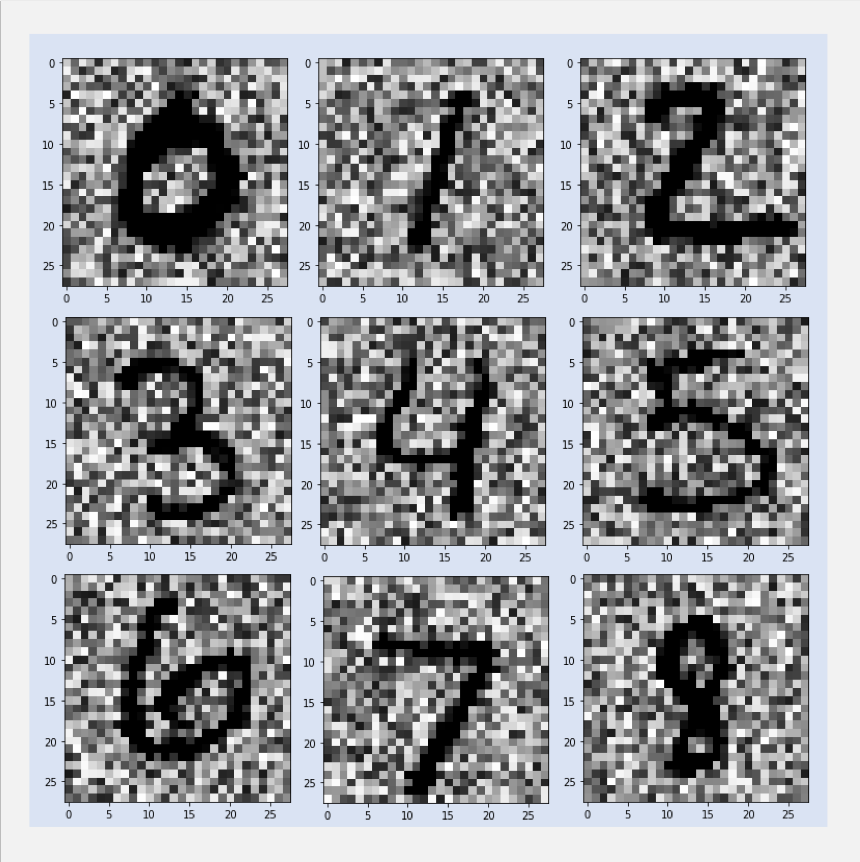
**

**Figure S3.** Samples with correct classification

**
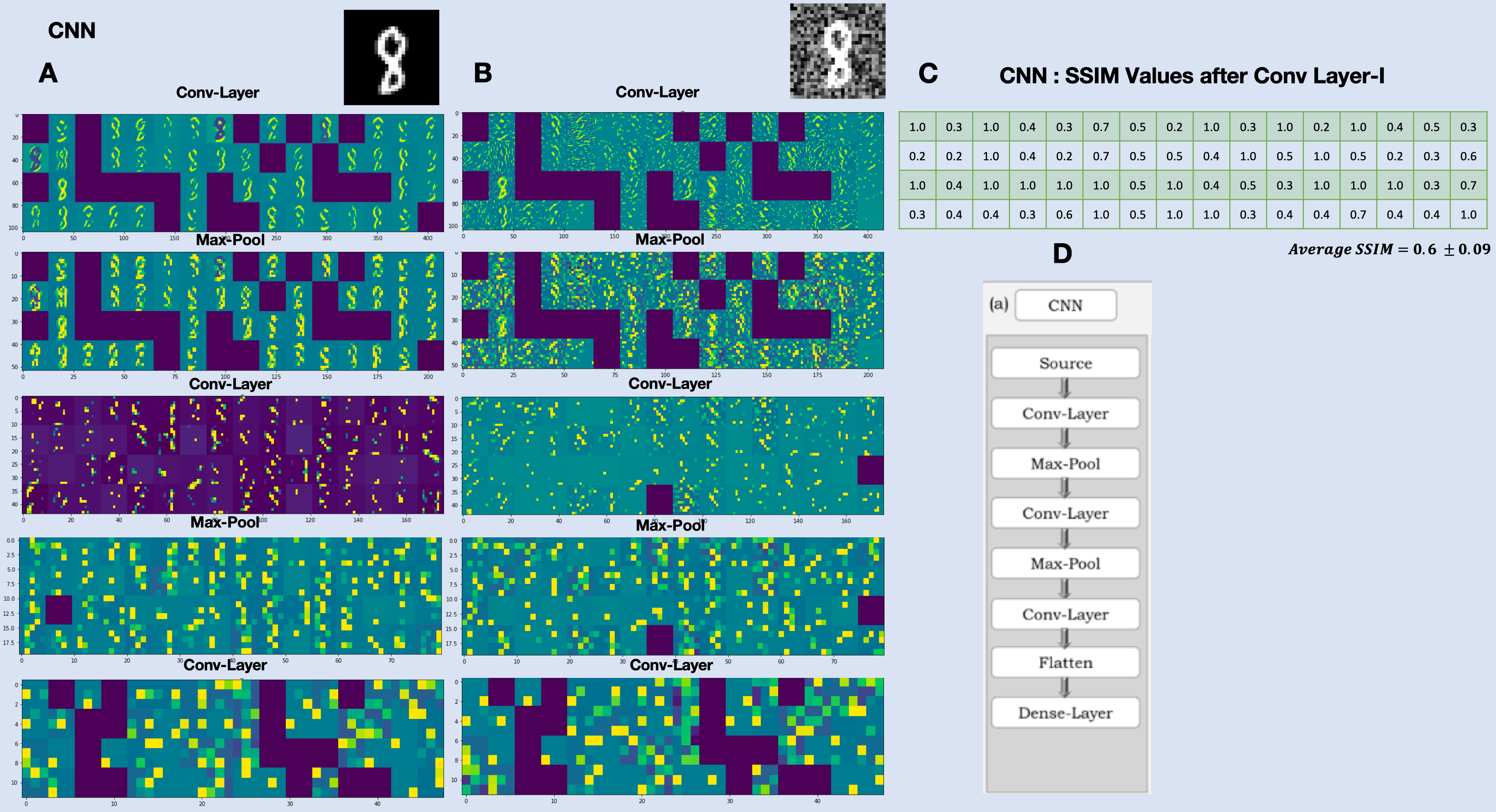
**

**Figure S4. Filtered output after different layers of standard CNN for clean and noisy images** (A) Filtered output after different layers of standard CNN for clean image (B) Filtered output after different layers of standard CNN for noisy image (C) SSIM values after Conv Layer-1 for standard CNN (D) Standard CNN architecture

**
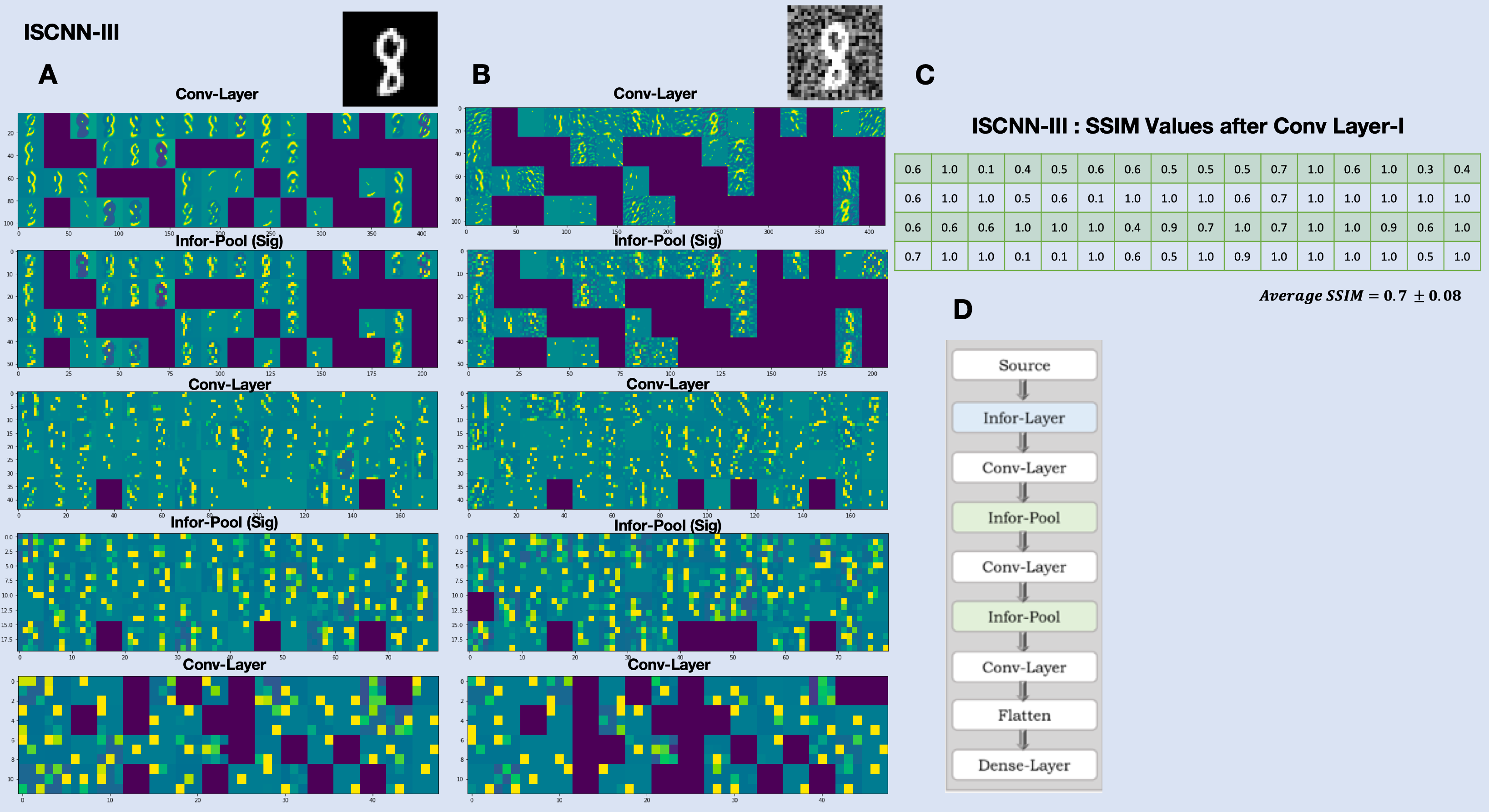
**

**Figure S5. Filtered output after different layers of ISCNN-III for clean and noisy images** (A) Filtered output after different layers of ISCNN-III for clean image (B) Filtered output after different layers of ISCNN-III for noisy image (C) SSIM values after Conv Layer-1 for ISCNN-III (D) ISCNN-III architecture


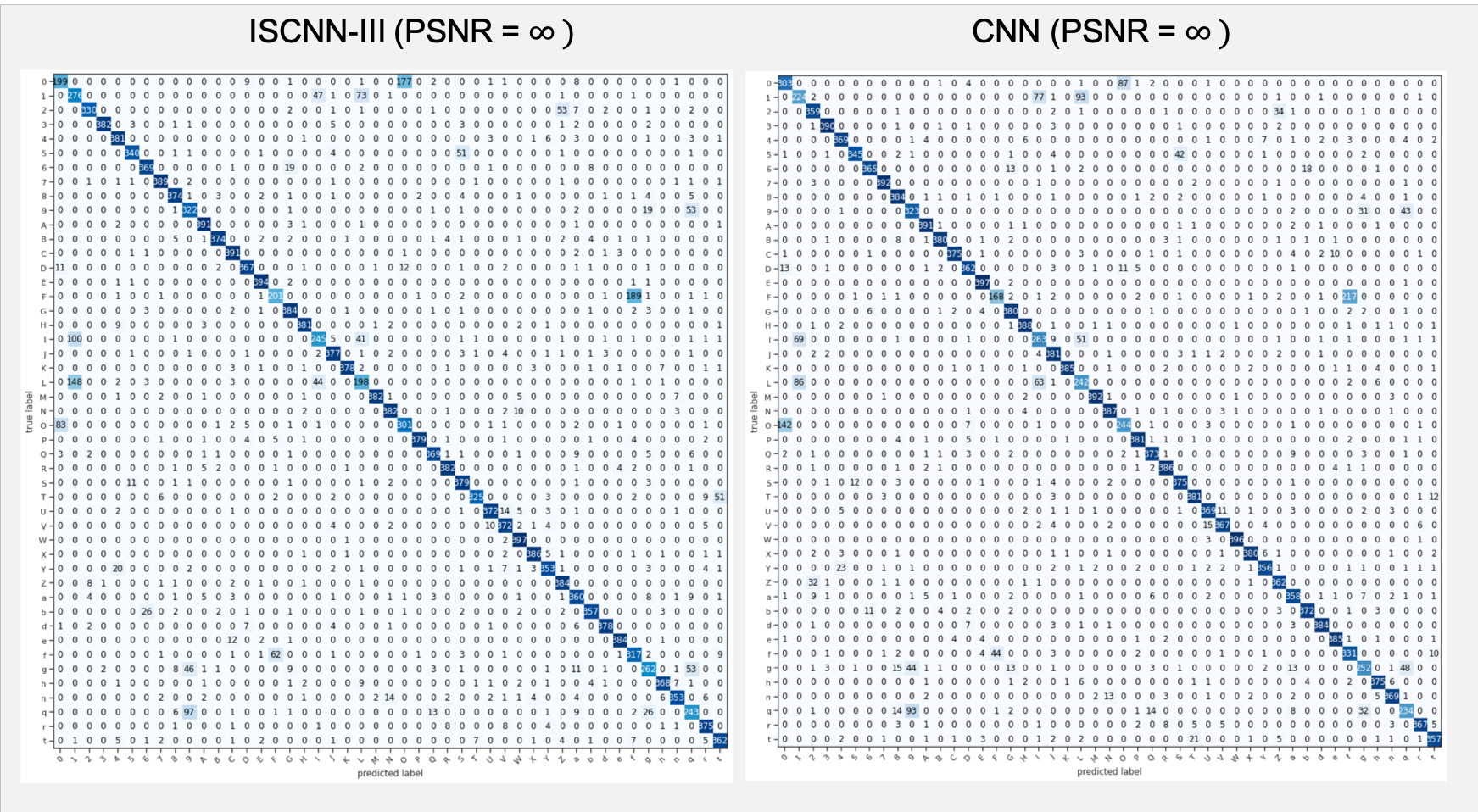

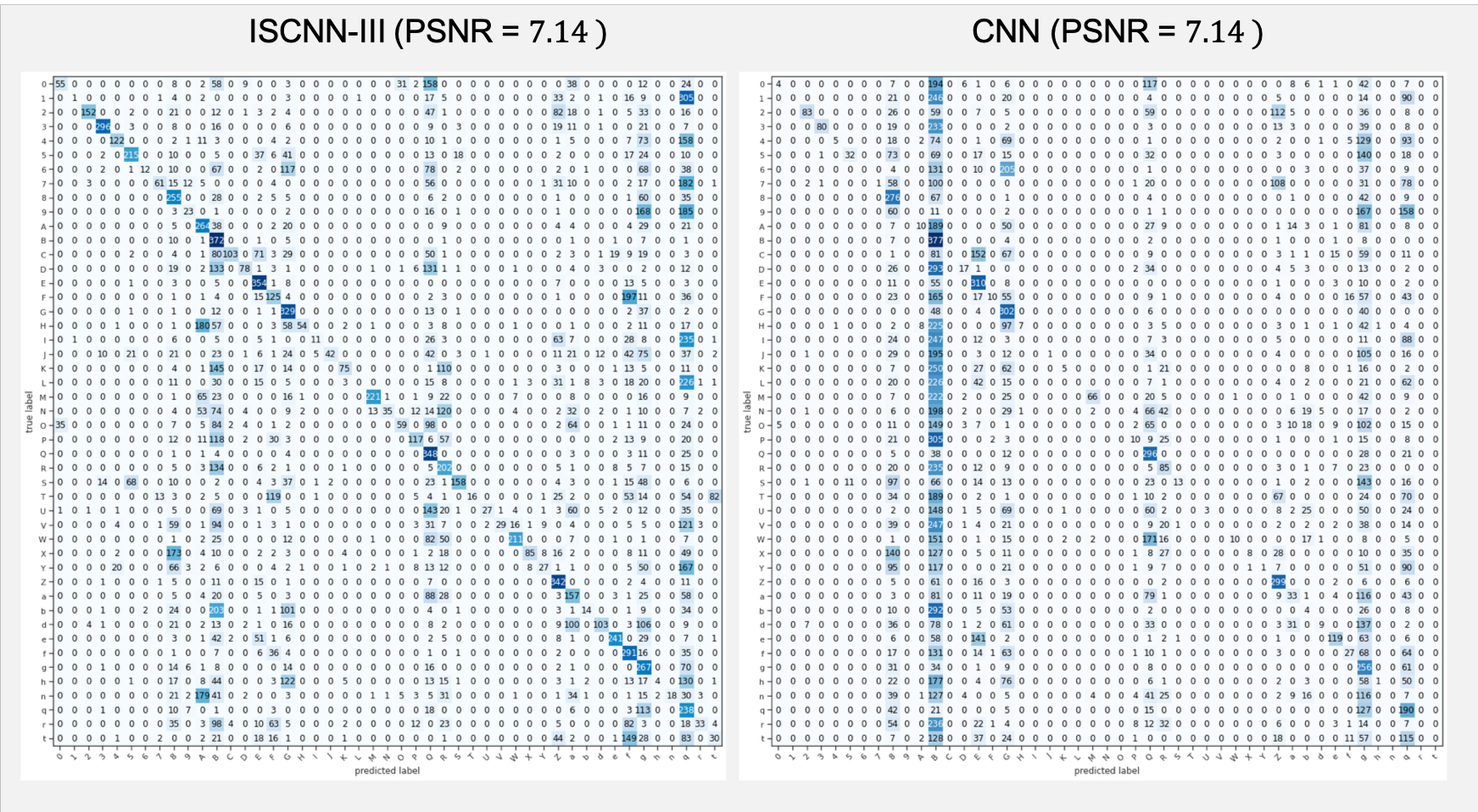


**Figure S6.** Confusion matrices for ISCNN-III and CNN for varying PSNR value for EMNIST dataset

**Supplementary Tables:**

**Table S1** explains the significant differences between the proposed models and the most relevant conventional DL models in design and functionality.

**Table S1.** Comparison of ISDL capabilities with the most relevant existing methods

| **Design - Functionality** | **DL-NN** | **DL-CNN** | **IS-NN, ISCNN** | **Difference specifics** |
| --- | --- | --- | --- | --- |
| Incorporation of the Information Set based layers | **✗** | **✗** | **✓** | ISDL models are equipped with infor-set layers and infor-set-pooling layers; peer models do not. |
| Extraction of higher-level encoded features in noise | **✗** | **✗** | **✓** | ISDL models extract efficient information from noisy data; peer methods often need additional pre-processing units |
| Computational efficiency | **Low** | **Low** | **High** | ISDL is computationally efficient higher than peer techniques since it includes information set layers. Still, peer methods often require additional pre-processing units, which decreases computational efficiency as sample sizes increase. |
| Auto-noise-suppression | **✗** | **✗** | **✓** | ISDL models perform auto-noise suppression, Figure 2, Figure S4, and Figure S5; peer methods do not. |
| Only need to be trained on clean samples | **✗** | **✗** | **✓** | ISDL models are trained only on clean samples; peer methods are often trained on a large number of diverse samples (clean and noisy). |
| High classification accuracy on noise samples | **✗** | **✗** | **✓** | ISDL models perform more effectively than conventional models on noisy samples. Figure 2, Figure 3B, Figure 4, Figure S4, Figure S5, Table 2, Table 6, Table 7 |
| Number of Training Parameters | Same | Same | Same | Both ISDL and conventional models have an equal number of training parameters |
| Number of hyperparameters | Less | Less | More | ISDL models require an additional hyperparameter to select as a gain function |

**Table S2.** Experimental Designs (EMNIST) : Infor-Set Based Convolutional Neural Networks

| **LAYERS** | **CNN** | **ISCNN-I** | **ISCNN-II** | **ISCNN-III** |
| --- | --- | --- | --- | --- |
| Layer-1 |  |  | Infor-Layer (Sig) | Infor-Layer (Sig-Exp) |
| Layer-2 | Conv (Relu) | Conv (Relu) | Conv (Relu) | Conv (Relu) |
| Layer-3 | Max Pool | Infor-Pool (Sig) | Max Pool | Infor-Pool (Sig) |
| Layer-4 | Conv (Relu) | Conv (Relu) | Conv (Relu) | Conv (Relu) |
| Layer-5 | Max Pool | Infor-Pool (Sig) | Max Pool | Infor-Pool (Sig) |
| Layer-6 | Flatten | Flatten | Flatten | Flatten |
| Layer-7 | Dense (Relu) | Dense (Relu) | Dense (Relu) | Dense (Relu) |
| Layer-8 | Dense (Relu) | Dense (Relu) | Dense (Relu) | Dense (Relu) |
| Layer-9 | Dense (Softmax) | Dense (Softmax) | Dense (Softmax) | Dense (Softmax) |

**Table S3** Layer details of CNN and ISCNN models for EMNIST dataset

| **Conventional CNN** | | **ISCNN-I** | | **ISCNN-II** | | **ISCNN-III** | |
| --- | --- | --- | --- | --- | --- | --- | --- |
| **Layer** | **Output Shape** | **Layer** | **Output Shape** | **Layer** | **Output Shape** | **Layer** | **Output Shape** |
| Input Layer | [(None, 28, 28, 1)] | Input Layer | [(None, 28, 28, 1)] | Input Layer | [(None, 28, 28, 1)] | Input Layer | [(None, 28, 28, 1)] |
| Conv-Layer (2D) | [(None, 28, 28, 32)] | Conv-Layer (2D) | [(None, 28, 28, 32)] | Infor-Layer (Sig) | [(None, 28, 28, 1)] | Infor-Layer (Sig-Exp) | [(None, 28, 28, 1)] |
| Max-Pool (2D) | [None, 14, 14, 32] | Infor-Layer (Sig) | [(None, 28, 28, 1)] | Conv-Layer (2D) | [(None, 28, 28, 32)] | Conv-Layer (2D) | [(None, 28, 28, 32)] |
| Conv-Layer (2D) | [(None, 10, 10, 48)] | Conv-Layer (2D) | [(None, 10, 10, 48)] | Max-Pool (2D) | [(None, 14, 14, 32)] | Infor-Pool (Sig) | [(None, 14, 14, 32)] |
| Max-Pool (2D) | [None, 5, 5, 48] | Infor-Layer (Sig) | [(None, 28, 28, 1)] | Conv-Layer (2D) | [(None, 10, 10, 48)] | Conv-Layer (2D) | [(None, 10, 10, 48)] |
| Flatten | [None, 1200] | Flatten | [None, 1200] | Max-Pool (2D) | [(None, 5, 5, 48)] | Infor-Pool (Sig) | [(None, 5, 5, 48)] |
| dense_1 | [None, 256] | dense_1 | [None, 256] | Flatten | [None, 1200] | Flatten | [None, 1200] |
| dense_2 | [None, 84] | dense_2 | [None, 84] | dense_1 | [None, 256] | dense_1 | [None, 256] |
| dense_3 | [None, 47] | dense_3 | [None, 47] | dense_2 | [None, 84] | dense_2 | [None, 84] |
|  |  |  |  | dense_3 | [None, 47] | dense_3 | [None, 47] |
|  |  |  |  |  |  |  |  |

**Evaluation Metrics for Multi-class Classification**

The confusion matrix is associated with most of the evaluation metrics used in the manuscript. The confusion matrix is a table with four distinct combinations of predicted and actual values, as shown below:


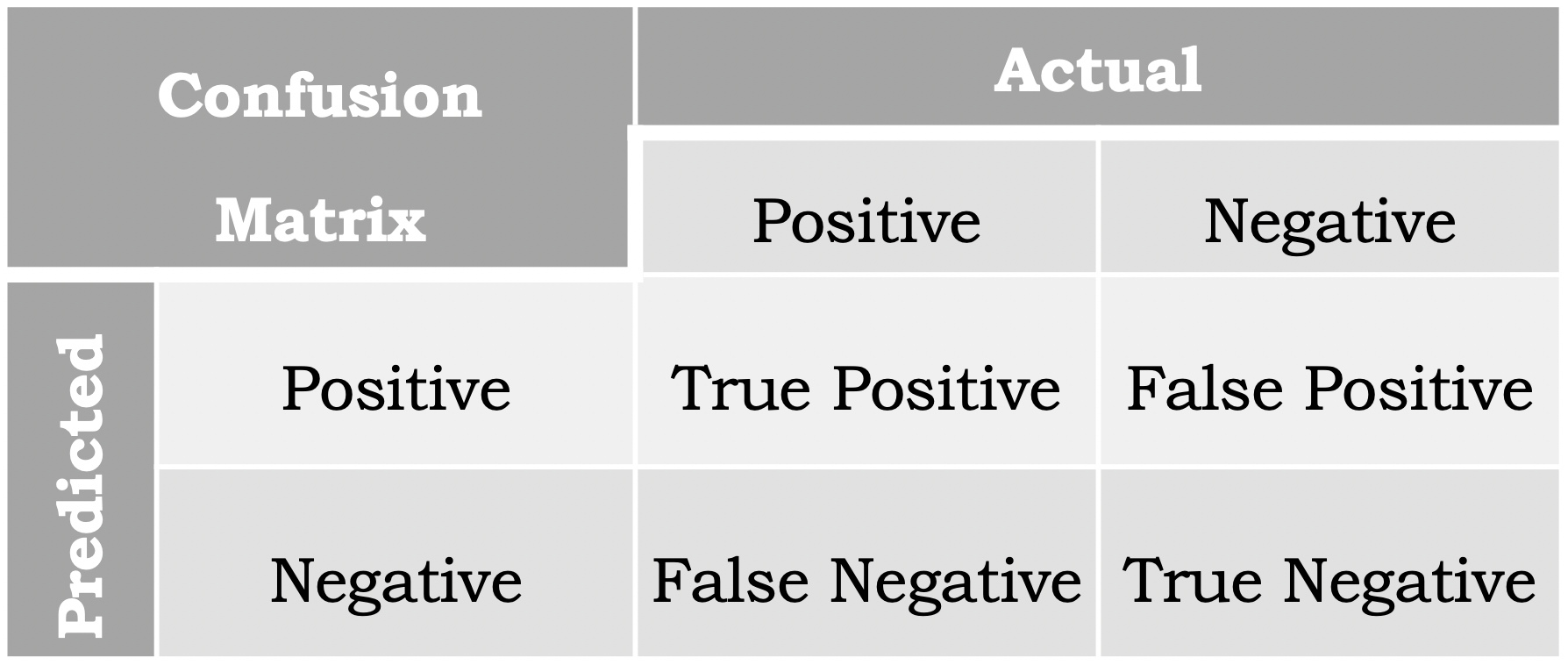


**True Positive:** Number of Positive, accurate predictions

**True Negative:** Number of Negative, accurate predictions

**False Positive:** Number of Positive false predictions

**False Negative:** Number of Negative false predictions

**Accuracy:** The accuracy parameter indicates how efficiently the model predicts the entire data set from both positive and negative classes. It is desirable to have a high accuracy value.

$$Accuracy= \frac{TP+TN}{TP+TN+FP+FN}$$

**Precision:** The number of classes that are truly positive out of all the predicted positive classes is represented by this parameter. It is desirable to have a high precision value.

$$Precision= \frac{TP}{TP+FP}$$

**Recall:** Recall is the percentage of actual positives that were correctly identified. It is desirable to have a high recall value.

$$Accuracy= \frac{TP}{TP+FN}$$

**F1 Score:** It is difficult to compare two models with high recall but a low precision. F-score can be used to evaluate precision and recall at the same time.

$$F1 Score= \frac{2 \times Precision\times Recall}{Precision+Recall}$$

**ROC-AUC Score**: The True Positive Rate (TPR) and False Positive Rate (FPR) are represented on the ROC curve's y-axis and x-axis, respectively. The area under the ROC curve is defined as the ROC-AUC score.
